# Hormones, psychotic disorders, and cognition in perinatal women: a mini review

**DOI:** 10.3389/fpsyt.2023.1296638

**Published:** 2024-01-05

**Authors:** Bruma Palacios-Hernández, Gabriela Ramírez-Alvarado, Bernarda Téllez-Alanís, Ana Luisa Lino-González, Miriam Penagos-Rivera, Adela Hernández-Galván

**Affiliations:** ^1^Cuerpo Académico “Cognición y Afectos” (UAEMor CA-81), Centro de Investigación Transdisciplinar en Psicología, Universidad Autónoma del Estado de Morelos, Cuernavaca, Mexico; ^2^Escuela de Estudios Superiores del Jicarero, Universidad Autónoma del Estado de Morelos, Jojutla de Juárez, Mexico; ^3^Facultad de Psicología, Universidad Autónoma del Estado de Morelos, Cuernavaca, Mexico; ^4^Investigación Biomédica Neurociencias Clínica, Instituto Nacional de Rehabilitación Luis Guillermo Ibarra Ibarra, Tlalpan, Mexico

**Keywords:** psychosis, hormones, cognition, pregnancy, parturition, postpartum period

## Abstract

Previous scientific evidence has shown a relationship between hormones and the onset and relapse of perinatal psychotic disorders (PPD) in women during pregnancy, childbirth, and the postpartum period. In healthy women the interaction between hormones and cognitive changes has been confirmed mainly in memory, attention, and executive function during pregnancy and postpartum, which respond to adaptive demands related to parenting tasks. In women with psychotic episodes there is a significant impairment in several cognitive functions, but studies of the perinatal period are limited. The objective of this mini review is to analyze the main findings to identify whether hormonal changes interact with the onset of PPD and cognitive impairment in perinatal women. The studies included samples of women with psychosis, risk of developing psychosis, bipolar psychosis, schizoaffective psychosis, and psychotic symptoms, during pregnancy and postpartum. Findings contributed to knowledge about five hypotheses regarding the relationship between hormones in the perinatal period and the appearance of PPD. Nevertheless, this review did not find reports of evidence of a relationship between hormonal production and cognitive function among women with clinically diagnosed PPD, suggesting a research gap. Clinical implications of assessing hormonal production and cognitive function in PPD are discussed. Although the evidence identified is scarce and heterogeneous, the findings call for further research with clinical samples on the role of hormones in perinatal psychotic disorders, especially as they relate to the study of cognition. This will promote more consistent evidence and understanding of PPD etiopathology that can guide early and effective multidisciplinary interventions.

## Introduction

The perinatal period is characterized by important hormonal changes. During pregnancy, there is an increase in levels of estrogen, progesterone, human placental lactogen, and human chorionic hormone (1). An increase in oxytocin triggers labor; it remains elevated during childcare and is an important factor in the mother-infant bonding. The level of estrogen declines after childbirth, and prolactin increases with lactation (1). These changes have effects in mood, cognition (2), and mental health related to “baby blues,” perinatal depression (1), and perinatal psychosis, the least studied of such phenomena.

The prevalence of schizophrenia is similar in men and women, but its expression is different. Women have greater premorbid functioning, different symptomatology, and a better course of illness (3). Postpartum psychosis (PP) affects behavior, mood, and cognition; its prevalence is 1–2 per 1,000 women (4), and its etiopathology is still unclear (5). It presents risks to the mother and baby, with a high risk of suicide, neglect of the infant, and, to a lesser extent, infanticide (6). Studies have explored hypotheses regarding the role of estrogen (6–8), thyroid hormones (9, 10), and cortisol (11) in the appearance of perinatal psychotic disorders (PPD) and such disorders outside the perinatal period. Estrogen has been reported to have a protective effect due to its action on the main brain neurotransmitters (dopamine, serotonin, and glutamate). Its alteration is associated with cognitive deficits, pathophysiology, and psychiatric disorder symptoms (12, 13).

Cognitive changes have been reported in healthy women during and after pregnancy, especially deficits in verbal learning and memory (2). Studies have found significant alterations in verbal fluency, sustained attention, alertness, verbal working memory, and social cognition after first psychotic episodes (14), although there has been less attention to the perinatal period.

The objective of this mini review is to identify whether hormonal changes interact with the onset of PPD and cognitive impairment in perinatal women.

## Search strategy

**Figure 1:**
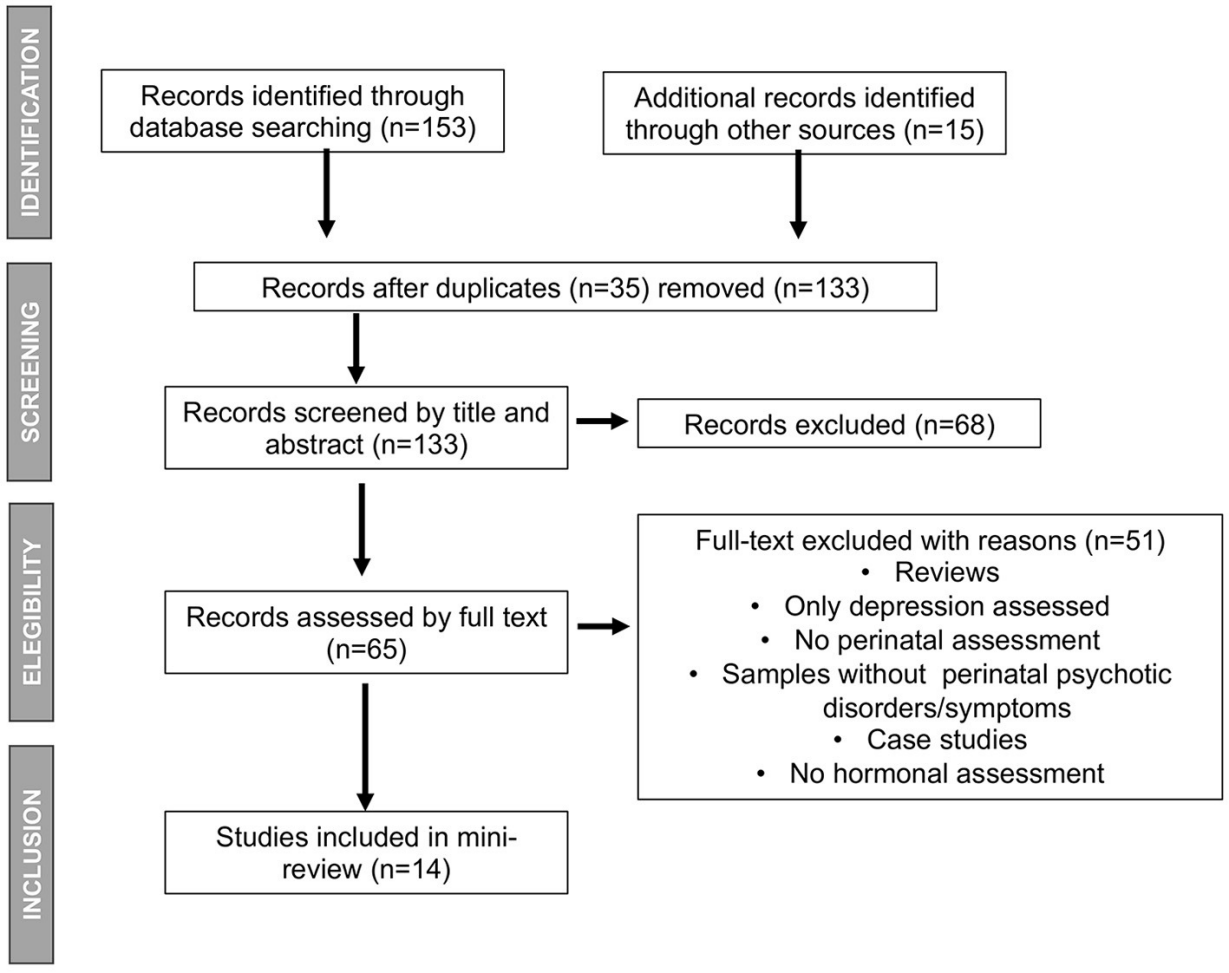
Mini review flowchart.

A search was conducted for empirical studies that analyzed hormones and their relationship with PPD and cognition in women, published in English and Spanish in the following databases through June 2023: PsycInfo, Medline, PsycArticle, ScienceDirect, SciELO, and Redalyc. The search keywords were *psychosis, hormones, pregnancy, parturition, postpartum period*, and *cognition/cognitive*. A complementary search was performed for similar studies in Research Gate and in the references listed in the publications identified. Inclusion criteria incorporated studies of women with psychosis, risk of developing psychosis, bipolar psychosis, schizoaffective psychosis, and psychotic symptoms during pregnancy and the first year of postpartum. Animal trials, case reports, case series, and review articles were excluded. A total of 133 studies were found, and 14 empirical studies met the inclusion criteria (Figure 1).

## Results

The findings contributed to knowledge mainly about the relationship between hormones in the perinatal period and the appearance of PPD. Little evidence was reported of a relationship between hormonal changes and cognitive function in perinatal women with psychosis.

Next, we present an analysis of the hypotheses suggested by the studies and the clinical implications of the evidence.

### Hormonal production and its relationship with the appearance of PPD

Findings of 14 studies (4, 13, 15–26) included five hypotheses regarding the relationship between hormones and the presence of PPD (Table 1): (H-I) changes in dopamine receptors, (H-II) increase in cortisol, (H-III) changes in estrogen levels, (H-IV) presence of thyroid dysfunction, and (H-V) decrease in oxytocin.

**Table 1:** Studies evaluating the relationship between hormones and presence, or risk of PPD organized by hypotheses^*^.

**Authors**	**PPD group**	**Control group**	**Measurements of hormones/others**	**Results**
**Hypothesis I: Changes in dopamine receptors**.				
Wieck et al. (15) England	Subsample of 15 pregnant women with previous history of psychosis (risk group).	Subsample of 15 pregnant women.	GH (blood) response to a subcutaneous injection of APO to observe the sensitivity of DA receptors on postpartum day 4.	Eight women from the PP risk group relapsed with a higher GH concentration in response to APO than those who did not relapse.
Meakin et al. (16) England	Subsample of 10 women at high risk of developing PP.	Subsample of 34 healthy multiparous women.	GH reaction to subcutaneous injection of APO to assess dopamine receptor sensitivity on days 4/5, 11/12 and 6 weeks postpartum. Serum total GH, progesterone, estradiol, and prolactin measurements.	No identified markers for the development of postpartum psychosis, neither in GH response to APO dose nor in circulating hormones.
Wieck et al. (17) England	Eight women recovered from a postpartum bipolar psychotic episode.	Nine women without psychiatric history.	GH response to APO injection to assess dopamine receptor sensitivity, after recovery from a psychotic episode in follicular and luteal phase.	Increase in GH concentration after APO administration in the luteal phase in women with a history of bipolar psychosis.
**Hypothesis II: Increase in cortisol**.				
Aas et al. (19) England	14 women with PP and 16 at risk of PP (history of bipolar or schizoaffective psychosis).	26 healthy women recruited from perinatal services.	Cortisol in saliva. They also measured several immunological markers in blood, including hs-CRP, TNFα, IL-4; IL-6.	Women with PP had higher cortisol levels at 30 min after waking than control participants. Hs-CRP was also higher in women with PP than in those at risk and in controls.
Hazelgrove et al. (20) England	51 women at risk of PP from pregnancy to postpartum.	61 healthy women, from pregnancy to postpartum.	Cortisol in saliva and inflammatory markers in blood: IL-1β, IL-2, IL-6, IL-8, TNFα, VEGF, EGF or hsCRP.	At 4 weeks postpartum, 22 women in the high-risk PP group relapsed. Logistic regression analysis indicated that severe childhood maltreatment and high cortisol levels in the third trimester of pregnancy were predictors of a relapse.
**Hypothesis III: Changes in estrogen levels**.				
Ahokas et al. (13) Finland	10 women with PP.	Without a control group (each woman acted as her own control).	Serum estradiol concentration from morning blood samples between 7 and 9 am before the first 17β-estradiol sublingual dose and weekly during 6 weeks of treatment.	Baseline levels of estradiol were lower than the threshold value for gonadal failure in all patients. During treatment the levels increased in nearly all women, and they approached the values normally found during follicular phase. Psychotic symptoms almost disappeared in week 2 of treatment.
Kumar et al. (23) United Kingdom	12 pregnant women with hypomania, mania or schizoaffective disorder who relapsed after childbirth.	17 pregnant women with hypomania, mania, or schizoaffective disorder without relapses after childbirth.	Three transdermal dose regimens of estrogen 17β-estradiol in three doses: 200, 400, and 800 μg/day. Fourth day after starting estradiol therapy (before relapse occurred), it was measured the sensitivity of the tubero-infundibular and dopaminergic system (plasma prolactin and GH response to APO).	Estradiol at all doses did not reduce the rate of relapse. No differences in neuroendocrine responses to apomorphine were detected between women receiving the high-dose and the lower-dose regimens.
Middle et al. (21) England	231 persons with bipolar psychosis. Subsample of parous women, 112 with a postpartum episode and 50 without episode.	110 non-psychiatric participants (32 men 78 women).	Variations of the ERα gene.	Did not find involvement of any ERα variations in the etiology of bipolar disorder or postpartum psychotic bipolar episodes.
Bendix et al. (22) Finland	10 women with PD and 4 PP.	28 women in postpartum.	Blood measurement, before and after sublingual estradiol dose. Measurement of allopregnanolone, progesterone, and the ratio of allopregnanolone to progesterone.	Allopregnanolone decreased during estradiol treatment. None of the investigated steroids (allopregnanolone, progesterone, estradiol) were associated with psychiatric symptoms before or after mood stabilization.
**Hypothesis IV: Presence of thyroid dysfunction**.				
Bergink et al. (25) Netherlands	31 primiparous women with PP.	117 pregnant women with postpartum follow-up.	Thyroid hormones (TSH and free thyroxine) in blood. Antithyroid peroxidase antibodies to detect ATID.	At 4 weeks postpartum, 19% of women with PP were identified as having ATID, compared to 5% of control participants.
**Hypothesis V: Decrease in oxytocin**.				
Ortega et al. (26) Spain	22 women with a first episode of psychosis during pregnancy.	20 healthy pregnant women.	Expression of genes and proteins for oxytocin, oxytocin receptor, vasopressin, and vasopressin receptor in placental tissue.	Women who had a first episode of psychosis during pregnancy showed increased gene and protein expression of oxytocin, vasopressin, and their receptors. This placental increase is explained as a compensatory effect.
**Two hypotheses**				
Buckwalter et al. (18) USA *Hypothesis II and III*	No group.	19 pregnant women with postpartum follow-up.	Serum levels of estradiol, progesterone, testosterone, DHEA, and cortisol. Two evaluations, 2 months before and 2 months after delivery. This is the only study that also evaluated cognitive processes.	During pregnancy, DHEA, progesterone, and testosterone correlated with psychotic symptoms, but not with cortisol. After delivery, neither estradiol, progesterone nor cortisol correlated with psychotic symptoms in mood measures.
Isik et al. (4) Türkiye *Hypothesis II and IV*	23 hospitalized women with PP.	30 age-matched control women.	TSH, free T4, free T3, cortisol, and other sex hormones (prolactin, FSH, LH, and DHEAS in blood).	Functional T3 levels were significantly lower in the PP group than in the control group. No significant differences were found in the other measured hormones.
Khedr et al. (24) Egypt *Hypothesis III and IV*	60 women with PP.	30 women without PP.	Measured levels of estrogen, progesterone, and thyroid hormones T3 free, T4, and TSH	A higher percentage of thyroid dysfunction (18%) and a significantly lower mean estrogen level were recorded in women with PP, as well as estrogen levels correlated with the total score of the Psychiatric Rating Scale.

^*^The articles are presented by hypothesis, and three of them studied two hypotheses simultaneously. PPD, Perinatal Psychotic Disorders; PP, Postpartum psychosis; GH, Growth hormone; APO, Apomorphine; DA, Dopamine; hs-CRP, High-sensitivity C-reactive protein; TNFα, Tumor Necrosis Factor; IL, Interleukin; VEGF, Vascular Endothelial Growth Factor; EGF, Epidermal Growth Factor; ERα, Estrogen receptor alpha; PD, Postpartum depression; TSH, Thyroid-stimulating hormone; ATID, Autoimmune thyroid disease; DHEA, Dehydroepiandrosterone; T3, Triiodothyronine; T4, levothyroxine; FSH, follicle-stimulating hormone; LH, Luteinizing hormone; DHEAS, sulfate of DHEA.

An increase in dopaminergic response is associated with the presence of psychosis and schizophrenia in men and women (27). This first hypothesis is explored in three studies (15–17) with the application of subcutaneous doses of the dopamine agonist apomorphine and the measurement of its effect on postpartum growth hormone (GH). Two studies analyzed whether the secretion of GH in greater quantities was related to effective binding of apomorphine in the dopaminergic receptors in the perinatal period. The first study (15) evaluated pregnant women: 29 at risk of postpartum psychosis (RPP) and a control group of 47. Fifteen participants from each group were selected for hormone testing and a dose of apomorphine on the fourth day postpartum. Eight of the 15 with RPP presented a relapse and a greater response of GH to apomorphine than the control group and the other seven women with RPP, suggesting that PPD is associated with greater sensitivity of the hypothalamic receptors and possible other regions of the brain.

A second study (16) evaluated ten mothers with high RPP (histories of manic psychosis) and compared them to mothers not at risk. They received three doses of apomorphine in the postpartum, and their progesterone, estradiol, and prolactin were measured. In contrast to the first study (15), no marker of GH response or any hormone was identified for the development of PPD. A third study (17) explored the role of the hypothalamic dopaminergic receptors by evaluating the GH response to apomorphine in the follicular and luteal phases of the menstrual cycle, comparing the effects in eight women who had recovered from postpartum bipolar psychotic episodes with those in nine women not at risk for PPD. This study identified a significant increase in the area under the curve of GH concentration after luteal phase administration of apomorphine in women with bipolar psychoses, suggesting a possible biological marker for the disorder, but one that must be considered with caution given the small sample size.

The second hypothesis posits the influence of cortisol in the appearance of psychosis (11) with a stress-vulnerability model analyzed by three studies (18–20). Buckwalter et al. (18) studied the relationship of mood, cortisol, and various hormones in 19 healthy women in pregnancy and postpartum. During pregnancy, dehydroepiandrosterone (DHEA), progesterone and testosterone were correlated with psychotic symptoms but not blood cortisol, though this was correlated with other aspects of mood. After childbirth, neither estradiol, progesterone, nor cortisol were correlated with any of the tests of mood. The study concluded that steroid hormones appear to play a role in psychotic symptoms during and after pregnancy, although it did not include participants with diagnosed mental disorders.

Aas et al. (19) studied the effect of cortisol and immunological markers associated with psychotic episodes in 14 women with RPP and with postpartum psychosis (PP), as compared with 26 healthy women. They found that women with PP had higher levels of cortisol 30 min after waking in the morning than those in the control group. The level of high-sensitivity C-reactive protein (hsCRP) was greater in women with PP, and they presented hypothalamic-hypophyseal-adrenal hyperactivity associated with a greater frequency of recent stress events, greater perceived stress, higher diurnal levels of cortisol and hsCRP than the control group. Hazelgrove et al. (20) had similar findings with a group of 51 women with RPP, of whom 22 had relapses. They measured cortisol in saliva, inflammatory blood markers, and psychosocial stress (severe child abuse and stressful life events). The group with PP relapses showed diurnal cortisol levels greater than those in the healthy group, and there were no differences between groups in inflammatory markers (IL-1β, IL-2, IL-6, IL-8, TNFα, VEGF, EGF, and hsCRP). Severe child abuse and high cortisol level in the third trimester of pregnancy predicted relapse at 4 weeks postpartum in women with RPP, after adjustment for clinical and sociodemographic covariables. Both studies (19, 20) supported the hypothesis of a relationship between elevated cortisol levels and the appearance of PP, and their association of stressful life events during postpartum also allows for direct inferences between variations in cortisol and RPP.

The third hypothesis proposes a relationship between variations in estrogen levels and the appearance of PPD, with six studies presenting contrasting evidence (13, 18, 21–24). Estradiol was not associated with postpartum psychotic symptoms in healthy mothers (18). Another study (21) examined the frequency of previously reported (28) four genetic variations of the estrogen receptor alpha (ERa) implicated in the appearance of PP. A mapping of 231 persons with bipolar disorder included 112 mothers who had a postpartum psychotic episode and 50 healthy mothers. The results showed no evidence that a variation in ERa was involved in the etiology of bipolar disorder or in the precipitation of perinatal psychotic bipolar episodes. Three studies analyzed the effect of estrogen treatment in preventing or reducing PPD episodes (13, 22, 23). One study (22) evaluated whether 4-week sublingual estradiol treatment reduced symptoms of depression and psychosis in ten women with postpartum depression and four with PP, and whether it affected levels of allopregnanolone and progesterone. Contrary to expectations, levels of allopregnanolone were reduced during treatment. None of the steroids investigated was associated with psychiatric symptoms before or after the women's mood was stabilized. A second study (13) presented evidence of a satisfactory response to treatment with sublingual 17β-estradiol in 10 women with PP, in whom estrogen deficiency was documented before treatment. The increase in blood estradiol levels in the first week of treatment was accompanied by a rapid and significant decrease in psychiatric symptoms, which practically disappeared in week 2, except for one woman who discontinued treatment. A third study (23) reported that treatment with transdermal 17β-estradiol at three different doses was not sufficient to achieve a prophylactic or therapeutic effect in 29 pregnant women with a history of psychosis and a high risk of relapse in postpartum, 12 of whom relapsed despite starting treatment within 48 h of birth. More recently, Khedr et al. (24) identified significantly lower levels of estrogen in 60 women with PP. The estrogen levels correlated negatively with the total score on the Psychiatric Rating Scale.

The fourth hypothesis relates to the influence of thyroid hormones on the appearance of PP and is reported in three studies (4, 24, 25). Khedr et al. (24) besides estrogen, also evaluated progesterone, and the thyroid hormones T3, T4, and TSH as factors in the appearance of PP in 60 women at 4 weeks postpartum. They found a greater proportion of thyroid dysfunction (18%) and the presence of transient thyroid dysfunction in women with PP. Bergink et al. (25) analyzed thyroid-stimulating hormone (TSH) and free thyroxine in 31 primiparous women with PP, comparing them with a control group of 177 healthy women. At 4 weeks postpartum they found that 19% of the women with PP developed autoimmune thyroid disease (AITD), vs. 5% of the control group, suggesting that this disease could be a risk factor for developing PP. A previous study (4) evaluated the thyroid hypothesis and the adrenal glands hypothesis (cortisol) about the etiology of PP. They recorded levels of sex hormones [prolactin, follicle-stimulating hormone, luteinizing hormone, and dehydroepiandrosterone (DHEA) sulfate] in the blood of 23 women with PP and 30 healthy postpartum women. Only the free T3 hormone showed significantly lower levels in the group with PP, but within normal levels in both groups. The authors suggest that there is a secondary thyroid effect in psychotic episodes, strengthening the hypothesis of a relationship between PP and thyroid alteration, vs. a possible causal association. These findings support the idea that psychotic episodes may interfere with the functioning of the hypothalamus-pituitary-thyroid axis, provoking a non-thyroid illness as a response to the underlying acute psychopathological process (4, 24).

The fifth and most recent hypothesis suggests a reduction in levels of oxytocin, in comparison to normal values in women with PPD, which was based on previous evidence from non-perinatal samples (29, 30). Only one study (26) met the criteria in our search. A non-perinatal study (29) found low levels of oxytocin in patients with a first psychotic episode, highlighting that increasing it can lessen alterations of mood and social cognition. Another study (30) reported that during menopause, lower levels of oxytocin in women with schizophrenia are associated with more severe psychiatric symptoms. However, the only study identified that focused on the perinatal period (26) did not support this hypothesis, which analyzed the expression of genes, proteins, oxytocin, arginine vasopressin (AVP), and their respective receptors in the placental tissue of 22 pregnant women after a first psychotic episode, in comparison with 20 healthy pregnant women. Those who had experienced the psychotic episode showed greater gene and protein expression of oxytocin and AVP and their receptors.

### Hormonal production and its cognitive effects on women with PPD

Only one study reported that hormones were related with cognition and perinatal psychotic symptoms in a healthy sample (18). Buckwalter et al. (18) measured serum levels of estradiol, progesterone, testosterone, cortisol, and DHEA, as well as cognitive processes, mood, and psychiatric symptoms in 19 healthy women 2 months before and after childbirth. During pregnancy, high levels of DHEA were associated with better visuospatial performance, verbal episodic memory, attention, better mood, and fewer psychotic symptoms. High cortisol was related to less perseverance in verbal learning. High progesterone correlated with psychotic symptoms and confusion. In the postpartum, high DHEA and cortisol were related with better cognitive performance. The decrease in DHEA after childbirth was associated with an increase in affective disorders and the increase of testosterone contributed to a worse mood, depression, and hostility. Cognitive deficits were greater during pregnancy regardless of mood, suggesting that extremely high levels of steroid hormones negatively affect cognition and mood, in addition to other perinatal factors. Although it has not been documented during pregnancy, cholinergic dysfunction would be consistent with the findings of this study.

Results from two studies located outside the initial search can indirectly suggest a relationship between hormones, cognition and PPD (31, 32) that requires further exploration. One study (31) in non-perinatal women with (*n* = 29) and without psychosis (*n* = 31) proposes that sex hormones have an activating effect on brain function during the menstrual cycle, and that circulating levels of estrogen in the brain influence cognition. Another study (32) identified that pregnant women who developed PP showed greater connectivity in the right dorsolateral prefrontal cortex and the ipsilateral middle temporal gyrus than the control group although significant differences between the groups were not identified.

### Clinical implications and intervention

The findings of the studies reviewed suggest the involvement of hormones in PPD and a relationship with cognitive deficits in healthy and non-perinatal samples, where exposure to psychosocial factors increases risk (19, 20). Interventions should seek improvement in mood and cognition to optimize decision making, learning, social cognition, and self-care, through promoting social support, sleep quality and strategies to reduce stress associated with affective and behavioral tasks related to the mother-child relationship.

There is evidence that during pregnancy a reduction in the gray matter of areas in the brain that involve social cognition relates with adaptive demands of motherhood (33). The findings are controversial as to whether pregnancy has negative consequences on brain physiology for middle-aged and older women by affecting the brain's response (plasticity or inflammation) (34). In postpartum depression, inflammation and oxidative stress can negatively affect neurons and promote degeneration (35). Therefore, cognitive assessments throughout the perinatal period could be useful for mothers at risk of PPD and severe perinatal mental health disorders.

The hypotheses analyzed provide some evidence on the effectiveness of hormonal treatments coupled with customary pharmaceutical approaches (antipsychotics, mood stabilizers), electroshock therapy (36–38), psychological and psychoeducational therapy, and family and institutional support (3). Nevertheless, caution is suggested since the evidence for them is not conclusive (36). The findings show a positive mood and cognitive impact of oxytocin (39, 40) and estradiol (13), but with emotional side effects (39).

Bolton (41) proposes the 4P model for care, which considers predisposing, precipitating, perpetuating, and protective factors where hormonal follow-up during the perinatal period in women at risk could help early identification of drastic changes related with the development of PPD and reduce its severity and/or relapses. Such follow-up would also allow for early intervention to minimize cognitive negative symptoms of the PPD and promote the affective and cognitive recovery of women with diverse clinical profiles of the perinatal psychotic disorders.

## Conclusions

There is evidence that suggests that hormones in women interact with PPD in the perinatal period. However, the evidence is insufficient about the relationship between hormones, PPD and cognitive impairments.

The findings regarding that hormones play a role in the appearance of PPD, suggested five hypotheses. Consistent evidence was found for three of those hypotheses: H-I, greater sensitivity of the hypothalamic receptors with an increased dopaminergic response; H-II, a stress-vulnerability model with elevated cortisol levels is related with PPD; H-IV, a relationship between PP and a thyroid dysfunction secondary to psychotic episodes. However, inconsistent findings were reported for the third and fifth hypotheses, suggesting caution and further exploration. The H-III, regarding a protective effect of estrogen, was only supported by two of six studies, although low estrogen levels have been associated with non-perinatal psychosis. The H-V, a decrease in oxytocin, was not associated with PPD in the only study identified by the search and reported increased expression of oxytocin in the placental tissue of pregnant women with the first episode of psychosis. Differences in methodologies, the heterogeneity of PPD, with the inclusion in the samples of women with risk for PPD and small sample sizes, as well as differences in methods of hormonal assessment limit the consistency of these findings. However, advances in the different hypotheses call for continued research to clarify the role of hormones in PPD in women and how that differs from non-perinatal psychotic disorders.

No studies reported about the role of hormones in cognition in women with clinically diagnosed PPD, and only one studied that role in a healthy sample. This represents an important research gap and highlights the need to distinguish between perinatal cognitive adaptive changes and pathological changes in the psychotic spectrum and the role of hormones on these processes. Longitudinal studies of primiparous mothers with clinically diagnosed PPD episodes conducted to observe the development of hormonal and cognitive changes could result in more consistent findings that can guide early treatment to minimize risk for mothers with PPD and their families. Finally, it is important that future studies include how these variables might interact with previous and current stressful life events, to provide a comprehensive biopsychosocial approach for the design of effective evidence-based multidisciplinary treatments.

## Author contributions

BP-H: Conceptualization, Investigation, Methodology, Project administration, Resources, Supervision, Writing—original draft, Writing—review & editing. GR-A: Conceptualization, Formal analysis, Investigation, Validation, Visualization, Writing—original draft. BT-A: Formal analysis, Methodology, Validation, Visualization, Writing—original draft. AL-G: Formal analysis, Investigation, Writing—review & editing. MP-R: Formal analysis, Investigation, Writing—review & editing. AH-G: Validation, Writing—review & editing.

## References

[1] Workman JL Barha CK Galea LA. Endocrine substrates of cognitive and affective changes during pregnancy and postpartum. Behav Neurosci. (2012) 126:54–72. 10.1037/a0025538 21967374

[2] Buckwalter JG Buckwalter DK Bluestein BW Stanczyk FZ. Pregnancy and postpartum: changes in cognition and mood. Prog Brain Res. (2001) 133:303–19. 10.1016/S0079-6123(01)33023-6 11589139

[3] González-Rodríguez A Guàrdia A Álvarez Pedrero A Betriu M Cobo J Acebillo S. Women with schizophrenia over the lifespan: health promotion, treatment and outcomes. Int J Environ Res Public Health. (2020) 17:5594. 10.3390/ijerph17155594 32756418 PMC7432627

[4] Isik M Ozdemir O Ucler R. Investigation of hormone levels in postpartum psychosis. Dusunen Adam J Psychiatr Neurol Sci. (2022) 35:48–55. 10.14744/DAJPNS.2022.00171

[5] Gale S Harlow BL. Postpartum mood disorders: a review of clinical and epidemiological factors. J Psychosom Obstet Gynaecol. (2003) 24:257–66. 10.3109/01674820309074690 14702886

[6] Perry A Gordon-Smith K Jones L Jones I. Phenomenology, epidemiology and aetiology of postpartum psychosis: a review. Brain Sci. (2021) 11:47. 10.3390/brainsci11010047 33406713 PMC7824357

[7] Brown JS Jr. Association of increased prenatal estrogen with risk factors for schizophrenia. Schizophr Bull. (2011) 37:946–9. 10.1093/schbul/sbp161 20053866 PMC3160212

[8] Brzezinski-Sinai NA Brzezinski A. Schizophrenia and sex hormones: what is the link? Front Psychiatry. (2020) 11:693. 10.3389/fpsyt.2020.00693 32760302 PMC7373790

[9] Misiak B Stańczykiewicz B Wiśniewski M Bartoli F Carra G Cavaleri D. Thyroid hormones in persons with schizophrenia: a systematic review and meta-analysis. Prog Neuropsychopharmacol Biol Psychiatry. (2021) 111:110402. 10.1016/j.pnpbp.2021.110402 34274416

[10] Sethy RR Garg S Ram D Tikka SK. Thyroid function in postpartum psychosis: an exploratory study. Asia Pac Psychiatry. (2021) 3:e12465. 10.1111/appy.12465 33742554

[11] Borges S Gayer-Anderson C Mondelli V. A systematic review of the activity of the hypothalamic-pituitary-adrenal axis in first episode psychosis. Psychoneuroendocrinology. (2013) 38:603–11. 10.1016/j.psyneuen.2012.12.025 23369532

[12] Hwang WJ Lee TY Kim NS Kwon JS. The role of estrogen receptors and their signaling across psychiatric disorders. Int J Mol Sci. (2020) 22:373. 10.3390/ijms22010373 33396472 PMC7794990

[13] Ahokas A Aito M Rimón R. Positive treatment effect of estradiol in postpartum psychosis: a pilot study. J Clin Psychiatry. (2000) 61:166–9. 10.4088/JCP.v61n0303 10817099

[14] Montaner-Ferrer MJ Gadea M Sanjuán J. Cognition and social functioning in first episode psychosis: a systematic review of longitudinal studies. Front Psychiatry. (2023) 14:1055012. 10.3389/fpsyt.2023.1055012 36950257 PMC10025326

[15] Wieck A Kumar R Hirst AD Marks MN Campbell IC Checkley SA. Increased sensitivity of dopamine receptors and recurrence of affective psychosis after childbirth. BMJ. (1991) 303:613–6. 10.1136/bmj.303.6803.613 1805821 PMC1671107

[16] Meakin CJ Brockington IF Lynch S Jones SR. Dopamine supersensitivity and hormonal status in puerperal psychosis. Br J Psychiatry. (1995) 166:73–9. 10.1192/bjp.166.1.73 7894880

[17] Wieck A Davies RA Hirst AD Brown N Papadopoulos A Marks MN. Menstrual cycle effects on hypothalamic dopamine receptor function in women with a history of puerperal bipolar disorder. J Psychopharmacol. (2003) 17:204–9. 10.1177/0269881103017002009 12870568

[18] Buckwalter JG Stanczyk FZ McCleary CA Bluestein BW Buckwalter DK Rankin KP. Pregnancy, the postpartum, and steroid hormones: effects on cognition and mood. Psychoneuroendocrinology. (1999) 24:69–84. 10.1016/S0306-4530(98)00044-4 10098220

[19] Aas M Vecchio C Pauls A Mehta M Williams S Hazelgrove K. Biological stress response in women at risk of postpartum psychosis: the role of life events and inflammation. Psychoneuroendocrinology. (2020) 113:104558. 10.1016/j.psyneuen.2019.104558 31923613

[20] Hazelgrove K Biaggi A Waites F Fuste M Osborne S Conroy S. Risk factors for postpartum relapse in women at risk of postpartum psychosis: the role of psychosocial stress and the biological stress system. Psychoneuroendocrinology. (2021) 128:105218. 10.1016/j.psyneuen.2021.105218 33892376

[21] Middle F Jones I Robertson E Morey J Lendon C Craddock N. Variation in the coding sequence and flanking splice junctions of the estrogen receptor alpha (ERalpha) gene does not play an important role in genetic susceptibility to bipolar disorder or bipolar affective puerperal psychosis. Am J Med Genet B Neuropsychiatr Genet. (2003) 118B:72–5. 10.1002/ajmg.b.10021 12627470

[22] Bendix M Bixo M Wihlbäck AC Ahokas A Jokinen J. Allopregnanolone and progesterone in estradiol treated severe postpartum depression and psychosis – preliminary findings. Neurol Psychiatry Brain Res. (2019) 34:50–7. 10.1016/j.npbr.2019.10.003

[23] Kumar C McIvor RJ Davies T Brown N Papadopoulos A Wieck A. Estrogen administration does not reduce the rate of recurrence of affective psychosis after childbirth. J Clin Psychiatry. (2003) 64:112–8. 10.4088/JCP.v64n0202 12633118

[24] Khedr EM Ramadan ES Osman MN Ahmed GK. Risk factors-related first episode postpartum psychosis among Egyptian women: the role of psychosocial and the biological factors. Egypt J Neurol Psychiatry Neurosurg. (2023) 59:51. 10.1186/s41983-023-00653-3

[25] Bergink V Kushner SA Pop V Kuijpens H Lambregtse-van den Berg MP Drexhage RC. Prevalence of autoimmune thyroid dysfunction in postpartum psychosis. Br J Psychiatry. (2011) 198:264–8. 10.1192/bjp.bp.110.082990 21343331

[26] Ortega MA García-Montero C Fraile-Martinez Ó De Leon-Oliva D Boaru DL Bravo C. Assessment of tissue expression of the oxytocin–vasopressin pathway in the placenta of women with a first-episode psychosis during pregnancy. Int J Mol Sci. (2023) 24:10254. 10.3390/ijms241210254 37373400 PMC10299487

[27] Kesby JP Eyles DW McGrath JJ Scott JG. Dopamine, psychosis and schizophrenia: the widening gap between basic and clinical neuroscience. Transl Psychiatry. (2018) 8:30. 10.1038/s41398-017-0071-9 29382821 PMC5802623

[28] Feng J Yan J Michaud S Craddock N Jones IR Cook EH. Scanning of estrogen receptor α (ERα) and thyroid hormone receptor α (TRα) genes in patients with psychiatric diseases: four missense mutations identified in ERα gene. Am J Med Genet A. (2001) 105:369–74. 10.1002/ajmg.1364 11378852

[29] Papadea D Dalla C Tata DA. Exploring a possible interplay between schizophrenia, oxytocin, and estrogens: a narrative review. Brain Sci. (2023) 13:461. 10.3390/brainsci13030461 36979271 PMC10046503

[30] Rubin LH Wehring HJ Demyanovich H Sue Carter C Pournajafi-Nazarloo H Feldman SM. Peripheral oxytocin and vasopressin are associated with clinical symptom severity and cognitive functioning in midlife women with chronic schizophrenia. Schizophr Res. (2018) 195:409–11. 10.1016/j.schres.2017.09.041 28965776

[31] Thompson K Sergejew A Kulkarni J. Estrogen affects cognition in women with psychosis. Psychiatry Res. (2000) 94:201–9. 10.1016/S0165-1781(00)00161-X 10889284

[32] Kowalczyk OS Pauls AM Fusté M Williams SCR Hazelgrove K Vecchio C. Neurocognitive correlates of working memory and emotional processing in postpartum psychosis: an fMRI study. Psychol Med. (2021) 51:1724–32. 10.1017/S0033291720000471 32174288

[33] Hoekzema E Barba-Müller E Pozzobon C Picado M Lucco F García-García D. Pregnancy leads to long-lasting changes in human brain structure. Nat Neurosci. (2017) 20:287–96. 10.1038/nn.4458 27991897

[34] Duarte-Guterman P Leuner B Galea LAM. The long and short term effects of motherhood on the brain. Front Neuroendocrinol. (2019) 53:100740. 10.1016/j.yfrne.2019.02.004 30826374

[35] Worthen RJ Beurel E. Inflammatory and neurodegenerative pathophysiology implicated in postpartum depression. Neurobiol Dis. (2022) 165:105646. 10.1016/j.nbd.2022.105646 35104645 PMC8956291

[36] Doucet S Jones I Letourneau N Dennis CL Blackmore ER. Interventions for the prevention and treatment of postpartum psychosis: a systematic review. Arch Womens Ment Health. (2011) 14:89–98. 10.1007/s00737-010-0199-6 21128087

[37] Sharma V Mazmanian D Palagini L Bramante A. Postpartum psychosis: revisiting phenomenology, nosology, and treatment. J Affect Disord Reports. (2022) 10:100378. 10.1016/j.jadr.2022.100378

[38] Jairaj C Seneviratne G Bergink V Sommer IE Dazzan P. Postpartum psychosis: a proposed treatment algorithm. J Psychopharmacol. (2023) 37:960–70. 10.1177/02698811231181573 37515460 PMC10612381

[39] Mah BL Van IJzendoorn MH Smith R Bakermans-Kranenburg MJ. Oxytocin in postnatally depressed mothers: its influence on mood and expressed emotion. Prog Neuropsychopharmacol Biol Psychiatry. (2013) 40:267–72. 10.1016/j.pnpbp.2012.10.005 23085508

[40] Donadon MF Martin-Santos R L Osório F. Oxytocin effects on the cognition of women with postpartum depression: a randomized, placebo-controlled clinical trial. Prog Neuropsychopharmacol Biol Psychiatry. (2021) 111:110098. 10.1016/j.pnpbp.2020.110098 32937192

[41] Bolton JW. Case formulation after engel-the 4P model: a philosophical case conference. Philos Psychiatr Psychol. (2014) 21:179–89. 10.1353/ppp.2014.0027

